# Map-based assessment of older adults’ life space: validity and reliability

**DOI:** 10.1186/s11556-020-00253-7

**Published:** 2020-11-28

**Authors:** Timo Hinrichs, Adriana Zanda, Michelle P. Fillekes, Pia Bereuter, Erja Portegijs, Taina Rantanen, Arno Schmidt-Trucksäss, Andreas W. Zeller, Robert Weibel

**Affiliations:** 1 Division of Sports and Exercise Medicine, Department of Sport, Exercise and Health, University of Basel, Birsstrasse 320 B, 4052 Basel, Switzerland; 2 Department of Geography, University of Zurich, Zurich, Switzerland; 3 University Research Priority Program “Dynamics of Healthy Aging”, University of Zurich, Zurich, Switzerland; 4 Institute of Geomatics Engineering, University of Applied Sciences and Arts, Northwestern Switzerland, Muttenz, Switzerland; 5 Faculty of Sport and Health Sciences and Gerontology Research Center, University of Jyvaskyla, Jyvaskyla, Finland; 6 Centre for Primary Health Care, University of Basel, Basel, Switzerland

**Keywords:** Geographic information systems, Environment, Mobility, Aged

## Abstract

**Background:**

Map-based tools have recently found their way into health-related research. They can potentially be used to quantify older adults’ life-space. This study aimed to evaluate the validity (vs. GPS) and the test-retest reliability of a map-based life-space assessment (MBA).

**Methods:**

Life-space of one full week was assessed by GPS and by MBA. MBA was repeated after approximately 3 weeks. Distance-related (mean and maximum distance from home) and area-related (convex hull, standard deviational ellipse) life-space indicators were calculated. Intraclass correlations (MBA vs. GPS and test-retest) were calculated in addition to Bland-Altman analyses (MBA vs. GPS).

**Results:**

Fifty-eight older adults (mean age 74, standard deviation 5.5 years; 39.7% women) participated in the study. Bland-Altman analyses showed the highest agreement between methods for the maximum distance from home. Intraclass correlation coefficients ranged between 0.19 (95% confidence interval 0 to 0.47) for convex hull and 0.72 (95% confidence interval 0.52 to 0.84) for maximum distance from home. Intraclass correlation coefficients for test-retest reliability ranged between 0.04 (95% confidence interval 0 to 0.30) for convex hull and 0.43 (95% confidence interval 0.19 to 0.62) for mean distance from home.

**Conclusions:**

While acceptable validity and reliability were found for the distance-related life-space parameters, MBA cannot be recommended for the assessment of area-related life-space parameters.

## Background

Life-space can be defined as the “spatial extent in which a person moves within a specified period” ( [1] , p. 155). It results from “the interaction between intrinsic capabilities of the person and the demands of the extrinsic environment” ([1], p. 155), including the physical, sociocultural, and the economic environment. In older adults, life-space is positively associated with social participation [2], quality of life [3], and physical activity [4, 5]. Limited life-space predicts disability in basic activities of daily living [6], falls and fractures [7], and mortality [8].

Until now, epidemiological studies mostly relied on questionnaires to measure life-space. These offer advantages such as low cost and time-efficiency. However, the geospatial information gained from questionnaires is rather coarse. As an example, the frequently used University of Alabama at Birmingham Study of Aging Life-Space Assessment assesses the subjective extent of an individual’s movement categorized into five spatial levels, ranging from the participant’s bedroom to places outside the participant’s home town [9]. Nowadays, global positioning system (GPS) technology offers the chance to objectively and much more precisely measure life space [10–12]. GPS devices allow tracking an individual’s location at high spatial and temporal resolution [13]. While high-end GPS devices can achieve decimetre accuracy, typical portable consumer GPS devices nowadays reach a positioning accuracy of 2–3 m under normal conditions [14]. However, barriers towards using GPS in large-scale studies include high costs and an elaborate handling of devices imposing a burden on participants; i.e., participants have to carry around the GPS-enabled device whenever they leave their home and the device’s battery has to be charged regularly [13]. A number of geographic approaches have been suggested aiming to quantify an individual’s life space based on GPS data, including the maximum distance from home [15], the area of convex hull [1, 16, 17], and the area of standard deviational ellipse [16, 18, 19] (see methods section). Additional qualitative information (e.g. on the purpose of an activity) cannot be captured directly from GPS, although, there are a number of health-related measures that can be derived from GPS tracking data, including use of transportation [20], time spent out of home [21], and exposure to certain environments (such as green spaces, fast-food restaurants or supermarkets [22]).

Questionnaire tools based on interactive digital maps, usually referred to as ‘Public Participation Geographic Information Systems’ [23] or ‘SoftGIS’ [24, 25], allow the combination of qualitative data (i.e. the “soft” knowledge produced by participants) with objective geographic information system data (e.g. street network, physical structure, building density etc.). In the context of our study, advantages of paper-based life-space questionnaires and GPS are combined: low cost, time efficiency, low burden on participants, and fine-grained spatial location information. Additional qualitative information, e.g. on the purpose of visiting a certain location, on the use of transportation modes, on the need for personal assistance or on environmental barriers and facilitators of mobility, may be collected simultaneously [24–27]. Map-based tools have recently found their way into health-related research [23, 25–28], but only since very recently are being used to quantify people’s life space [29].

For the present study, a questionnaire tool based on interactive digital maps aiming to assess older adults’ life-space was developed [30]. The map-based assessment (MBA) consisted of a retrospective evaluation of all places visited within the past 7 days and was assisted by a trained interviewer. It started with a short tutorial on how to navigate through the questionnaire and on how to mark locations on the map. In a first step, participants were asked to mark their home on the map. After that – following the example of the University of Alabama at Birmingham Study of Aging Life-Space Assessment – visited places as well as the frequency of visits were assessed in 3 consecutive steps referring to the three levels: 1) in the neighbourhood; 2) outside the neighbourhood, but within town; and 3) outside town.

The aim of this study was to evaluate the validity of MBA of older adults’ life space versus GPS. Furthermore, test-retest reliability of the MBA was investigated.

## Methods

### Study design and participants

This validity (cross-sectional) and reliability (test-retest) study was approved by the Ethics Committee Northwest/Central Switzerland (Reg.-No. 2016–01259). To be eligible for the study, subjects had to be community-dwelling, aged 65 or older, and be able to communicate appropriately. Subjects had to report that they regularly leave their home (≥ 3 times per usual week) and be able to walk short distances without the help of another person. Only those who were unable to visit the study centre were excluded. All participants provided written informed consent.

A convenience sample was recruited for this study. Potential participants were approached by study personnel in general practitioner practices as well as through adult education centres, clubs and service organizations for older adults. People who expressed interest in participating in studies at the corresponding author’s institution in the past were also approached.

Assessments took place in the study centre and in participants’ daily lives carrying a GPS tracking device. Assessments were conducted in four steps:

1. Baseline (study centre): assessment of basic participant characteristics; hand-out of GPS device.
2. Observation period (participants’ daily lives): one week of continuous ambulatory GPS measurement.
3. First follow-up (study centre): hand-in of GPS device; assessment of life space by MBA. The MBA referred to the past seven days, i.e. the period when GPS measurements took place.
4. Second follow-up (study centre; about 3 weeks after first follow-up): retest of MBA. Again, the MBA referred to the past seven days.

### Measures

#### Map-based assessment of life space

**Fig. 1:**
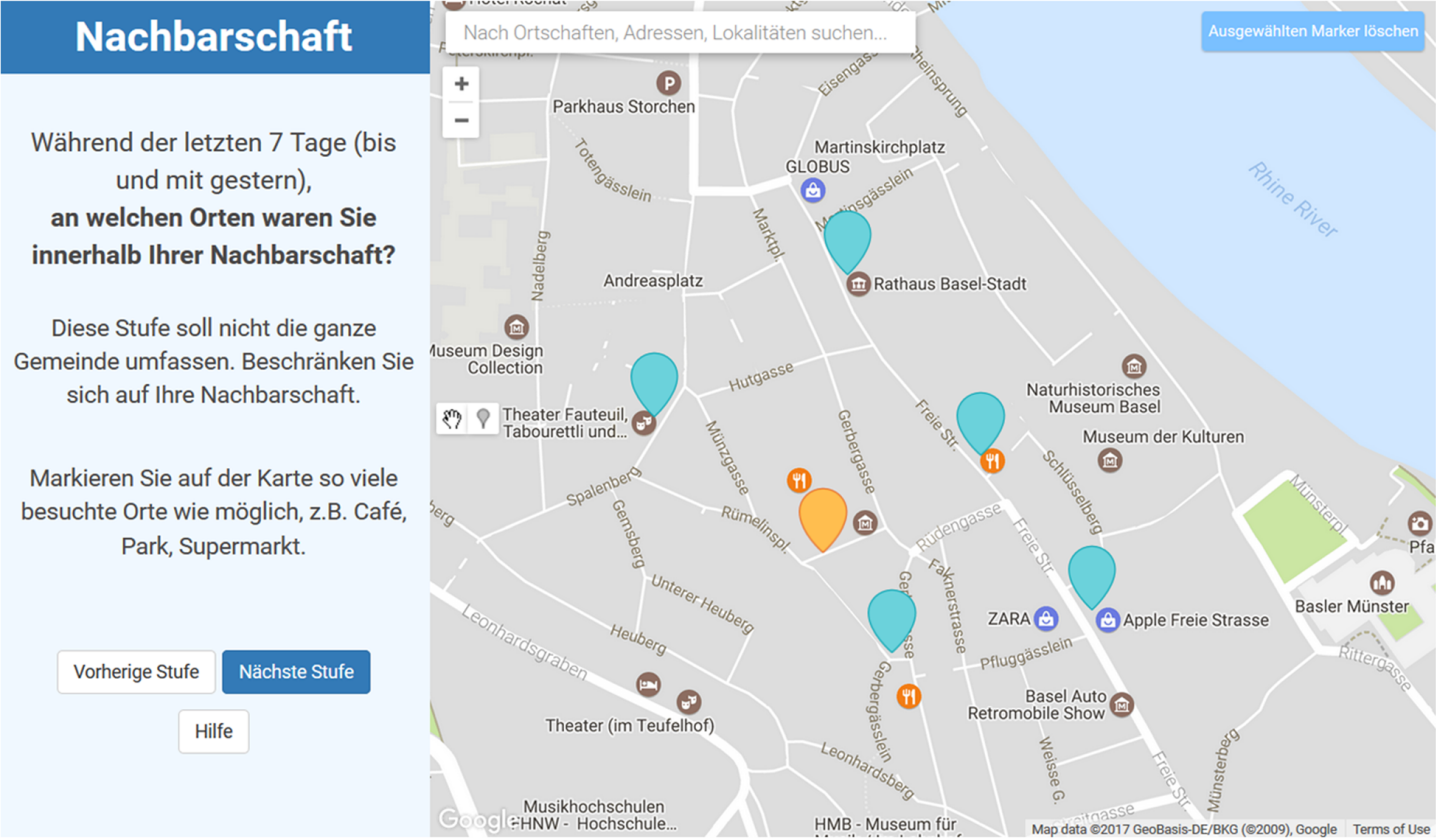
Screenshot of a fictitious life-space assessment by the interactive map-based assessment. It illustrates that the map application splits the screen into a left and right pane. The left side states questions, in the style of the following: “During the past 7 days (until yesterday), which places did you visit in your neighborhood?” The questions are supposed to be answered by marking points on the map on the right hand side of the screen. In this case, the (fictitious) participant marked his/her home in a previous step (orange marker) and five visited places in the neighborhood in the current step (blue markers). The map and its functionalities are created with the Google Maps JavaScript API and its Drawing library. The map design is a modified version of the Google Maps map, based on the Gowalla design from Snazzy Maps (Available at: https://snazzymaps.com/style/20/gowalla [Accessed November 1, 2020]).

The MBA consisted of a retrospective evaluation of outdoor activities of the past 7 days by a web-based questionnaire using digital geographical maps [30]. The questionnaire started with a short tutorial on how to mark (or delete) locations on the map and on how to navigate through the screens. The application splits the screen into a left and right pane with questions on the left and an interactive map on the right pane (Fig. 1). Whenever, a location was marked (by moving the cursor to the correct location on the map and clicking the left mouse-button), a pop-up window opened automatically and asked the participant for the frequency of visiting this location within the past 7 days. In case a marker was set to a wrong location, it could always be deleted or dragged to another location. A trained interviewer was always present and helped the participant in case of any problems, e.g. with handling the user interface or with understanding questions or required tasks. In the context of our study, major advantages of using digital maps (instead of paper maps) included: (a) the possibility to automatically search for addresses and specific locations (by typing the location’s name into a search bar), (b) the flexible scale of the map (participants could zoom in an out), additionally facilitating the identification of visited places and improving location accuracy, and (b) the simplified data handling with geographic coordinates of a location being directly saved to an electronic database. After the tutorial, participants were asked to mark their home on the map. After that, visited places and frequency of visits were assessed in 3 steps (one screen per step): 1) in the neighbourhood; 2) outside the neighbourhood, but within town; and 3) outside town.

The captured longitude and latitude coordinates were projected to a metric coordinate system [31]. The following previously suggested distance-related and area-related indicators of life-space were calculated: mean distance of visited locations from home (straight line distance) [1], maximum distance from home (straight line distance) [15], area of convex hull [1, 16, 17], and area of standard deviational ellipse [16, 18, 19]. The convex hull is the smallest convex polygon that encompasses all points of a point set [18]. The standard deviational ellipse measures the dispersion and orientation of a point set; we used the ‘one’-standard deviational ellipse containing 68% of all points. This life-space measure is less sensitive to outliers than the maximum distance and the convex hull [19, 32]. Calculations of mean distance and standard deviational ellipse were weighted for the frequency of visiting a location [19].

#### GPS-based assessment of life space

**Fig. 2:**
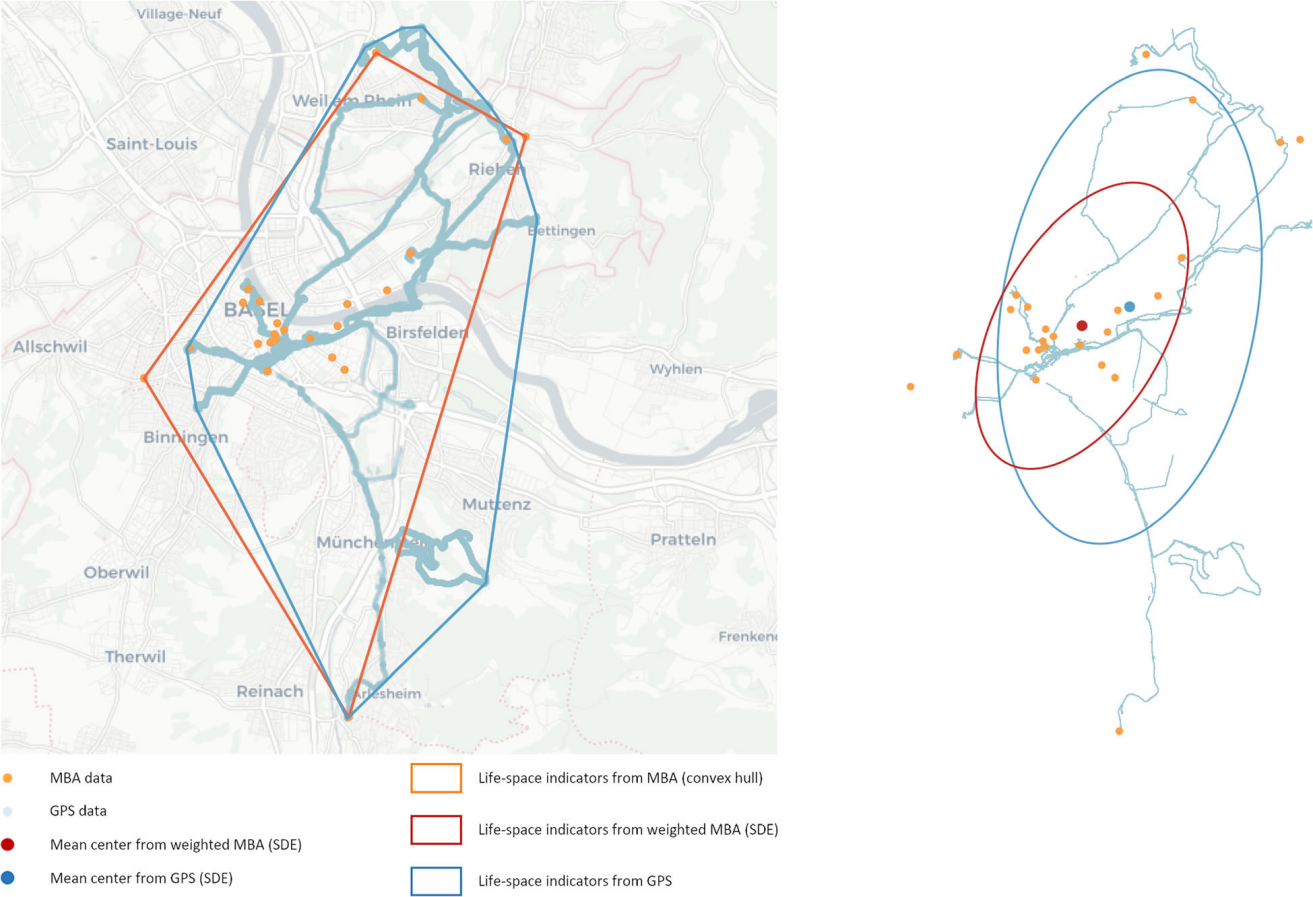
Illustration of different possibilities to quantify life space by area-related life-space indicators from GPS data (blue) as well as from data collected with the map-based assessment (orange): left – convex hull; right – standard deviational ellipse. Map-based assessment data were weighted by the number of visits per location for calculation of the standard deviational ellipse

During the observation period, participants wore a small portable GPS tracking device (uTrail, CDD Ltd., Athens, Greece) for 7 consecutive full days. Participants received instructions on correct device handling prior to the observation period and a phone call during the observation period to check for potential handling problems. Again, mean distance and maximum distance from home as well as convex hull and standard deviational ellipse were calculated. GPS data and data collected with MBA of an example participant are shown in Fig. 2; life space of the respective participant indicated by convex hull and standard deviational ellipse derived from GPS and MBA data are also illustrated in Fig. 2. GPS-based assessment of life space was considered as being valid, if the following criteria were fulfilled: minimum of 4 days with at least 9 h between the first and the last GPS fix of the day, including at least 2 weekdays and at least one weekend day [33–35].

#### Basic participant characteristics

Age, sex, living alone, living area (urban/suburban/rural) and availability of a private car were assessed by self-report. Education was measured as total years of formal education including school and vocational training [36]. Financial hardship was assessed by the question ‘did you experience financial difficulties that restricted your everyday life (participation) over the past four weeks?’; answer categories were ‘had no impact’, ‘has complicated my life somewhat’, and ‘has complicated my life massively’ [37]. Weight and height were measured by a trained assessor; body mass index was calculated. Frequency of falls and use of a walking aid were assessed by self-report [38]. Cognitive state was evaluated by the Mini-Mental State Examination [39].

### Statistical analyses

Participant characteristics were analysed descriptively. In order to evaluate the validity of MBA versus GPS, life-space parameters derived from MBA and GPS were assessed for agreement between methods by performing Bland-Altman-Analyses and by calculating intraclass correlations (type A,1) [40, 41]. As a measure of test-retest reliability of the MBA, intraclass correlations (type A,1) were calculated [41]. SPSS Statistics 24 (IBM Inc., Armonk, NY, USA) was used for statistical analyses; the level of significance was set at *p* ≤ 0.05.

### Sample size

Sample size calculation was based on the Bland-Altman-Analyses for agreement between MBA and GPS. Independent of the outcome parameter used, a sample size of 47 results in an accuracy of ±0.5*s for the estimation of limits of agreement, where *s* is the standard deviation of the differences between measurements by the two methods [40, 42]. Accounting for 15% of participants with invalid GPS measurements, the target sample size was 56.

## Results

### Participants

Fifty-eight older adults (mean age 74, standard deviation 5.5 years; 39.7% women) participated in the study (Table 1).

**Table 1:** Basic participant characteristics (*N* = 58)

Characteristic	Mean ± SD or n (%)	Min.; Max.
**Sociodemographics**		
Age, mean ± SD	74.0 ± 5.5	65; 87
Female, n (%)	23 (39.7)	
Living alone, n (%)	21 (36.2)	
Living area, n (%)		
Urban	29 (50.0)	
Suburban	22 (37.9)	
Rural	7 (12.1)	
Private car available, n (%)	45 (77.6)	
Years of education, n (%)	13.8 ± 3.1	8; 22
Financial hardship, n (%)		
No difficulties	55 (94.8)	
Some difficulties	2 (3.4)	
Severe difficulties	1 (1.7)	
**Health-related parameters**		
Body Mass Index, mean ± SD	25.4 ± 3.5	18.8; 34.3
Number of falls in past 12 months, n (%)		
0	41 (70.7)	
1	12 (20.7)	
≥ 2	5 (8.6)	
Use of walking aid in past week, n (%)		
No aid	57 (98.3)	
Walking stick	1 (1.7)	
MMSE Count (0–30), mean ± SD	27.6 ± 2.3	17; 30

*SD* Standard deviation; *MMSE* Mini Mental State Examination

### Validity

**Fig. 3:**
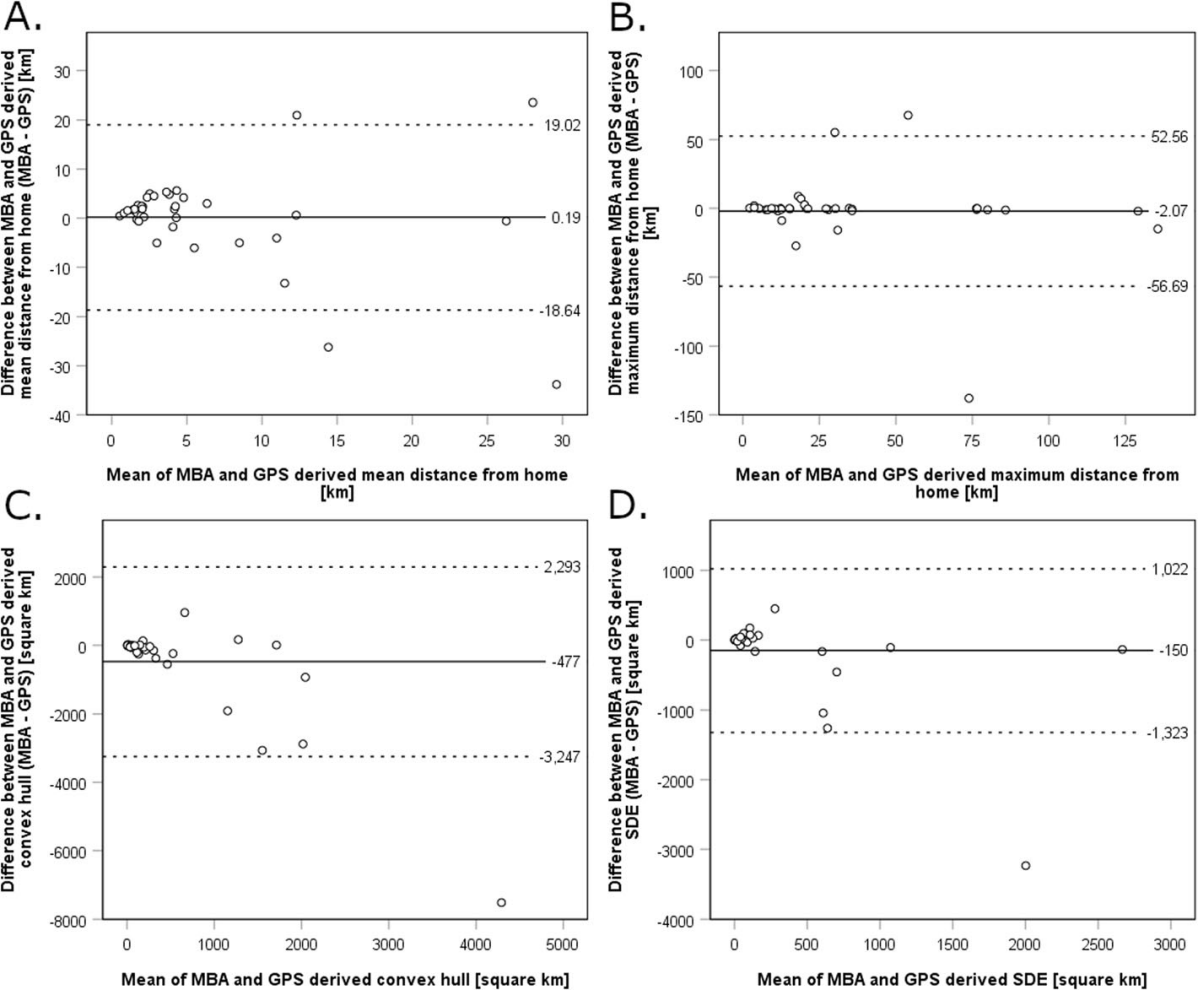
Bland-Altman plots illustrating the agreement of mean distance from home (**a**), maximum distance from home (**b**), convex hull (**c**) and standard deviational ellipse (SDE) (**d**) derived from the map-based assessment (MBA) and GPS measurements. The continuous horizontal line shows the mean difference between measurements by the two methods; the dashed lines show the limits of agreement, defined as the mean difference plus and minus 1.96 times the standard deviation of the differences

Twenty participants did not meet the above-mentioned criteria for a valid GPS-based life-space assessment; GPS data of another participant showed massive errors due to a broken device. Therefore, analyses of agreement between GPS and MBA refer to the remaining 37 participants. Those excluded had a higher mean age than those analyzed (76, standard deviation 5.1 years vs. 73, standard deviation 5.6 years) and they were more frequently female (50% vs. 35%). Bland-Altman-Analyses (Fig. 3) showed the highest agreement between methods for the maximum distance from home with a mean difference of − 2.07 km and narrow limits of agreement (Fig. 3, B). The agreement was lowest for convex hull area with a mean difference of − 477 km^2^, wide limits of agreement, and a tendency towards decreasing agreement with higher mean of methods (Fig. 3, C). This was also reflected by the intraclass correlation coefficients, ranging between 0.19 (convex hull; 95% confidence interval 0 to 0.47) and 0.72 (maximum distance from home; 95% confidence interval 0.52 to 0.84) (Table 2), the latter being interpretable as “substantial” according to Landis and Koch [43].

**Table 2:** Intraclass correlations between map-based assessment and the respective GPS parameters

	Map-based assessment of life space															
	Mean distance from home (km)				Maximum distance from home (km)				Convex hull (km^2^)				Standard deviational ellipse (km^2^)			
			95% CI				95% CI				95% CI				95% CI	
**GPS-based assessment of life-space**	**N**	**ICC**	**Lower**	**Upper**	**N**	**ICC**	**Lower**	**Upper**	**N**	**ICC**	**Lower**	**Upper**	**N**	**ICC**	**Lower**	**Upper**
Mean distance from home (km)	37	0.42*	0.11	0.66												
Maximum distance from home (km)					37	0.72*	0.52	0.84								
Convex hull (km^2^)									37	0.19	0	0.47				
Standard deviational ellipse (km^2^)													37	0.55*	0.29	0.74

*ICC* Intraclass Correlation Coefficient; *CI* Confidence Interval

* *p* ≤ .05

### Reliability

One participant dropped out between first and second follow-up. Another participant only marked one visited location on the map at second follow-up so that area-related parameters could not be calculated. Therefore, reliability analyses refer to 57 participants for mean and maximum distance from home and 56 participants for convex hull and standard deviational ellipse. Mean time period between the two MBAs was 22.3, standard deviation 7.9 days. Intraclass correlation coefficients are shown in Table 3; they were not statistically significant for standard deviational ellipse and convex hull [43].

**Table 3:** Test-retest reliability of the map-based assessment (first/second follow-up)

			95% CI	
Measure	N	ICC	Lower	Upper
Mean distance from home (km)	57	0.43*	0.19	0.62
Maximum distance from home (km)	57	0.26*	< 0.01	0.49
Convex hull (km^2^)	56	0.04	0	0.30
Standard deviational ellipse (km^2^)	56	0.14	0	0.38

*ICC* Intraclass Correlation Coefficient; *CI* Confidence Interval

* p ≤ .05

## Discussion

This study investigated the validity and reliability of an MBA of older adults’ life space. Distance-related and area-related life-space indicators were considered. The highest agreement between methods was found for the maximum distance from home. The highest test-retest reliability was found for the mean distance from home.

The present MBA assessed the life space retrospectively for a whole week. Therefore, the most important factor limiting agreement between the two methods was probably recall bias; i.e. participants did not remember all visited places. Similarly to Ullrich et al., who developed a modified version of the University of Alabama at Birmingham Study of Aging Life-Space Assessment for older persons with cognitive impairment [44], we limited the assessment period to one week in order to reduce this sort of bias. In our sample, only one participant had a Mini Mental State Examination count below 24 [45]; i.e. cognitive impairment was probably not a very relevant issue. A potential option to reduce recall bias in future map-based tools would be the daily (or even more frequent) assessment of visited places. This would however limit the possibility to provide personal assistance by a trained interviewer and participants would have to be provided with regular access to the tool. It seems that participants remembered the visited place with the furthest distance from home quite well, which was reflected by a high agreement between methods for this parameter. Even though this parameter only provides a very limited picture of a person’s life space, future studies using digital maps might choose to limit their life-space assessment to this single parameter.

Shareck et al. (2013) developed a map-based tool to obtain information on people’s ‘regularly visited locations’ [46]. Participants of their validation study were asked to provide details about the locations of predefined habitual activities. The convex hull areas (*n* = 23; mean age 37, standard deviation 12 years) derived from the MBA (median convex hull area 2.6 km^2^, interquartile range 1.0 to 6.3 km^2^) were smaller than those derived from an 8-day GPS tracking (median convex hull area 27.8 km^2^, interquartile range 11.7 to 186.9 km^2^). More recently, a study by Kestens et al. (2018) compared convex hull areas derived from a map-based tool (‘VERITAS’) with convex hull areas derived from 7-day GPS tracking [47]. Similar to the tool described by Shareck et al., the VERITAS questionnaire assesses destinations within predefined categories that participants visit regularly without specifying a particular recall period. Again, MBA and GPS convex hulls areas differed markedly (median convex hull area 33.0 km^2^, interquartile range 7.0 to 368.8 km^2^; and 147.9, IQR 50.3 to 1348.6 km^2^, respectively) (*n* = 234; mean age 57.8, standard deviation 11.6 years). The tendency towards finding smaller convex-hull areas by MBA compared to GPS seems to be in line with our results. However, the difference between MBA and GPS was much larger in our sample of older adults (mean difference 477 km^2^ for the convex-hull area). In conclusion, asking for ‘regularly visited locations’ within predefined activity categories (instead of all activities of the past week) might further contribute towards a reduction of recall bias.

Even though GPS measurements can be considered as ‘gold standard’ with respect to spatio-temporal accuracy of the data, they also have many defaults such as weak or no satellite signal or compliance issues (e.g. people not wearing or charging the devices). This may also limit the agreement between methods. As the GPS-based assessment was our ‘gold-standard’, we chose a rather strict definition of a ‘valid’ GPS measurement. This led to a larger number of invalid measurements than initially expected. However, a sample size of 37 generally still results in an accuracy of ±0.56*s for the estimation of limits of agreement in Bland-Altman Analyses [40]. The resulting widening of the limits of agreement as well as the widening of the 95% confidence intervals of the calculated intraclass correlation coefficients did not affect our conclusions. Sensitivity analyses with less strict definitions of a ‘valid’ GPS measurement did not markedly change the results.

Choosing a rather short recall period for the MBA of only one week might have contributed to the limited test-retest reliability. While the reliability was fair to moderate for the two distance-related life-space parameters, intraclass correlations did not reach statistical significance for the two area-related parameters. To explain the low reliability of the two area-related parameters, it has to be considered that the life space of two different weeks was assessed and especially in a highly functioning sample like ours (only one participant using a walking aid; only 5 participants with 2 or more falls in the past year), life space may vary significantly from week to week. In comparison, Baker et al. showed an intraclass correlation coefficient between test and retest (2-week follow-up) of the LSA composite score of 0.96 (95% confidence interval 0.95 to 0.97), which assesses life space of the past 4 weeks, takes the degree of independence into account and, in contrast to the MBA, includes in-house movement [9]. Ullrich et al. found an intraclass correlation coefficient between test and retest (2-day follow-up) for their modified 1-week LSA composite score of 0.91 (95% confidence interval 0.87 to 0.94) [44].

In conclusion, there is a tension between the need for lower recall durations – in order to minimize recall bias – and longer assessment periods – in order to minimize variability – which has to be considered by developers of future MBAs. As a first step, further research on the variability of life-space in older adults is needed. The optimal assessment period needed in order to reliably capture an older adults’ activity routine is still largely unknown. A recent GPS study suggested that, for working-age adults, the largely applied 7-day measurement period should at least be extended to 14 days for reliable estimates of an individual’s activity routine [22]. As a second step, possibilities to minimize recall bias should be explored. Considering the rapidly increasing proportion of smartphone users in the older population [48, 49], the implementation of an MBA into an easy-to-use smartphone application (with daily or even more frequent assessments) might be a feasible option for future studies. Making use of a smartphone application would even allow to combine the advantages of an MBA with the technological measurement properties offered by modern smartphones (including GPS and inertial measurement units) [12].

## Conclusions

This study contributes to the exploration of possibilities and limitations of using digital geographical maps in health-related research in older adults. Acceptable validity and reliability of the MBA were found for the distance-related life-space parameters. In the presented form, MBA cannot be recommended for the assessment of area-related life-space parameters, at least in a sample of highly functioning older people and for a recall period of only one week.

## Data Availability

The datasets used and/or analysed during the current study are available from the corresponding author on reasonable request.

## Abbreviations

GPS: Global Positioning System
MBA: Map-Based Assessment
